# Proximity ligation assay to study protein–protein interactions of proteins on two different cells

**DOI:** 10.2144/btn-2018-0049

**Published:** 2018-09-19

**Authors:** Rushikesh Sable, Nithya Jambunathan, Sitanshu Singh, Sandeep Pallerla, Konstantin G Kousoulas, Seetharama Jois

**Affiliations:** 1 Basic Pharmaceutical Sciences, School of Pharmacy, University of Louisiana at Monroe, LA 71201, USA; 2 Division of Biotechnology & Molecular Medicine & Department of Pathobiological Sciences, School of Veterinary Medicine, Louisiana State University, Baton Rouge, LA, USA

**Keywords:** assays, bioanalysis, flourescence

## Abstract

Protein–protein interactions (PPI) by homo-, hetero- or oligo-merization in the cellular environment regulate cellular processes. PPI can be inhibited by antibodies, small molecules or peptides, and this inhibition has therapeutic value. A recently developed method, the proximity ligation assay (PLA), provides detection of PPI in the cellular environment. However, most applications using this assay are for proteins expressed in the same cell. We employ PLA for the first time to study PPI of cell surface proteins on two different cells. Inhibition of PPI using a peptide inhibitor is also quantified using this assay; PLA is used to detect PPI of CD2 and CD58 between Jurkat cells (T cells) and human fibroblast-like synoviocyte-rheumatoid arthritis cells that are important in the immune response in the autoimmune disease rheumatoid arthritis. This assay provides direct evidence of inhibition of PPI of two proteins on different cell surfaces.

## METHOD SUMMARY

The proximity ligation assay (PLA) technique has been used to study protein–protein interactions (PPI) within a particular cell or to study the PPI of cell surface proteins that are expressed on the same cell. Here, for the first time, we report a PLA to be used on the surface of different cells. We have used this technique to visualize the interactions between proteins present on Jurkat (a nonadherent cell line) and HFLS-RA (an adherent cell line) cells in the same experiment. The assay was performed using the Duolink^®^II assay kit (Sigma Aldrich or Olink Bioscience, Uppsala, Sweden).

## Graphical abstract

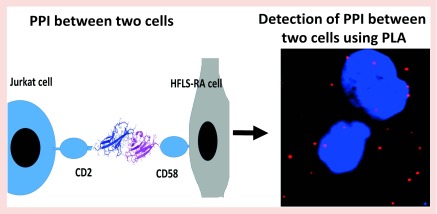

In almost all physiological processes, the medium for communication between two cells is facilitated by the interaction of proteins [1]. The up- or down-regulation of proteins and the deregulated interaction of these proteins usually lead to disease conditions. Rational drug designs targeting these protein–protein interactions (PPI) have gained much popularity in the past decade [2,3]. Hence, methods to study PPI and their inhibition is important. Many proteins interact at the cell surface to send signals inside the cell from the extracellular region, and PPI on the cell surface are targeted for drug discovery [4,5]. Epidermal growth factor receptor as well as CD2–CD58 proteins have been used for targeting cancer and immunomodulation [6,7]. Among the cell surface proteins that assist communication between cells, CD2–CD58 PPI plays a key role in T-cell signaling and has implications in autoimmune diseases such as rheumatoid arthritis (RA). Cyclic peptides that were modified for stability using sunflower trypsin inhibitor template (SFTI) previously used to target CD2–CD58 interaction [8] have been used as inhibitors of PPI in this study. It was hypothesized that these peptides will mimic the CD2 adhesion domain and will bind to CD58 protein, which ultimately will interrupt the CD2–CD58 PPIs. SFTI-a peptide binds to the CD58 protein and inhibits CD2–CD58 interaction. The inhibition of protein–protein interactions results in the modulation of immune response. It was reported that SFTI-a peptide was found to be a potent compound for inhibiting cell adhesion in the immune response in cell-based *in vitro* and *ex vivo* assays to suppress T-cell immune response [8]. It was therefore important to investigate whether the designed peptides inhibit the CD2–CD58 PPI by the mechanism we anticipated in the hypothesis. Detailed interaction between proteins CD2 and CD58 was elucidated by the crystal structure of CD2–CD58 complex (Figure 1A) [9]. There are ten salt bridges and five hydrogen bonds between the CD2 and CD58 adhesion domains and, although the interaction is relatively weak (Kd ∼1–10 μM), it is highly specific, making it an important interaction in the immune response.

**Figure 1.**
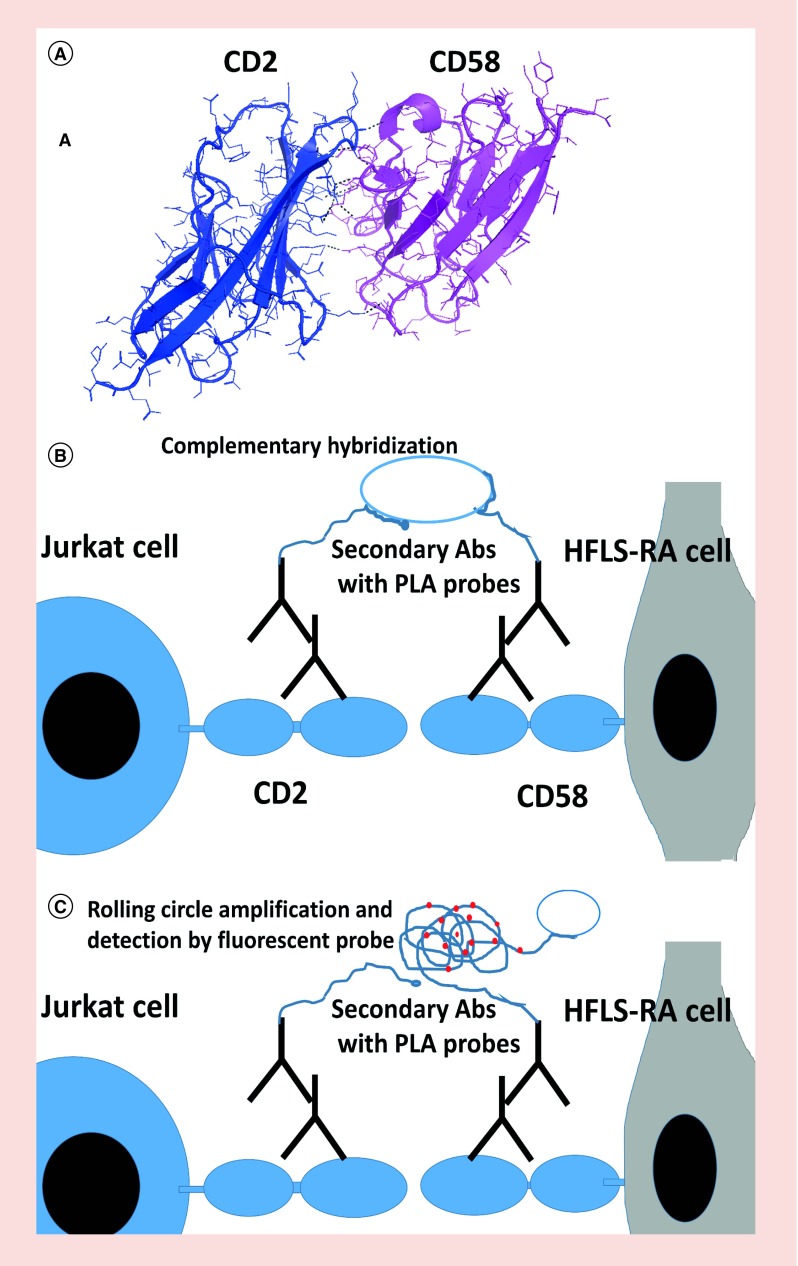
**Protein–protein interactions of CD2–CD58 and its detection using proximity ligation assay.** **(A)** Crystal structure of complex of CD2–CD58 (PDB ID: 1QA9) showing adhesion domain of proteins. **(B & C)** a schematic diagram of PPI between CD2 and CD58 from T cells and HFLS-RA cells and detection of PPI using PLA. HFLS-RA: Human fibroblast-like synoviocyte-rheumatoid arthritis; PLA: Proximity ligation assay; PPI: Protein–protein interactions.

Conventionally, coimmunoprecipitation with western blot technique is used to detect PPIs [10]. The proximity ligation assay (PLA) is a new powerful technique not only to visualize PPIs but also to quantify PPIs and their inhibition by small molecules, peptides and antibodies. Unlike traditional immunocytochemistry, which displays only co-localization of proteins, the PLA helps to detect and visualize PPIs using a fluorescence probe in a native state of the cells *in vitro* and in samples from *in vivo* studies [11–15].

In PLA, the PPI can be detected using primary antibodies and secondary antibodies/probes against the specific proteins participating in the PPI. The protein-specific primary antibodies act as binding sites for species-specific secondary antibodies/probes, which are attached to DNA oligonucleotides. When these PLA probes bind to the target and are within the required proximity (distance ≤ 40 nm), DNA ligation occurs, linking both PLA probes upon incubating with ligase. After addition of polymerase, the DNA-ligated circles will be amplified in numbers to which labeled complementary oligonucleotide probes will be added, and they will show bright red fluorescent spots. In short, we can visualize the PPIs using fluorescent probes [13]. To date, the researchers have successfully used the PLA technique to evaluate the PPI between two proteins present on the same cells [14]. Here, for the first time, we employed PLA to visualize the interaction between CD2 and CD58 proteins that are present on two different cells, Jurkat cells and human fibroblast-like synoviocyte-rheumatoid arthritis (HFLS-RA) cells, respectively.

In an effort to elucidate the entire protein network (interactome) of the human body, details of protein–protein interaction elucidation are important to obtain a global picture of biological processes in the body [1]. Deregulation of PPI is also important in human diseases. Thus, elucidating PPI between two cells using PLA helps to understand the cellular communication between the two cells. Furthermore, the inhibition of PPI by drug-like molecules or modulation of PPI can be quantified using this assay. Since antibodies are used for labeling particular proteins, the assay detects highly specific interactions. The assay also provides information on co-localization of proteins when the two cells make contact. Since immune cells make contact during immune response, this assay is useful for studying proteins involved in the immune network and complements the existing assays used to study protein–protein interactions at the immunological synapse [16,17]. A schematic diagram of the proposed PLA for proteins on different cells is shown in Figure 1B & C. CD2 is known to be expressed on T cells. CD58 is expressed on all epithelial cells but is known to be on antigen-presenting cells [18,19]. We used HFLS-RA cells as a model for antigen-presenting cells that express CD58. In rheumatoid arthritis, CD58 is known to be overexpressed, and this leads to recruitment of T cells at the joints, causing inflammation [20]. Thus, HFLS-RA cells that express CD58 serve as a good model for studying CD58 protein and its importance in the immune response.

## Materials & methods

### Cell lines/cells

T-leukemia Jurkat cell line was purchased from the American Type Culture Collection (MD, USA). The cells were maintained in RPMI1640 medium (with 10% FBS and 0.1 mg/ml penicillin/streptomycin). HFLS-RA cells were purchased from Cell Applications, Inc. (CA, USA) and were maintained in human synoviocyte medium provided by Cell Applications.

### Primary antibodies & PLA kit

Specific primary antibodies against CD58 (sc-6983, RRID: AB_2076111) c-terminus goat polyclonal and CD2 (sc-365017, RRID: AB_10707813) mouse monoclonal were purchased from Santa Cruz Biotechnology (TX, USA). PLA *in situ* red mouse/goat kit was purchased from Duolink/Sigma Aldrich (MO, USA). This kit includes mouse and goat secondary antibodies with probes, blocking solution, wash buffers A and B, amplification solution, ligase solution and detection reagent.

### Peptide synthesis

Synthesis of cyclic peptide SFTI-a, Cyclo(CKASAPPSCYDGDD), was carried out in three major steps: 1) Linear sequence synthesis with an automatic peptide synthesizer (Protein Technologies, Inc., AZ, USA); 2) head-to-tail cyclization; and 3) disulfide bond formation. For fluorescent labeling of peptide SFTI-a, Lys in the peptide sequence was replaced with Lys containing an azide side chain. The azide side chain was used to conjugate fluorescent label 4,4-difluoro-4-bora-3a,4a-diaza-s-indacene (BODIPY) [21], resulting in the conjugate Cyclo(CK-(BODIPY)-ASAPPSCYDGDD). The detailed procedure for synthesis of the peptide is described in our previous work [8]. Peptides SFTI-a and SFTI-a with lysine azide (N_3_) and fluorescence-labeled SFTI-a were purified by preparative HPLC (Waters Corp., MA, USA) using acetonitrile and water containing 0.1% TFA. Molecular weights of the synthesized peptide and the conjugate were confirmed by mass spectrometry. BODIPY was obtained from Dr Vicente, Department of Chemistry, Louisiana State University, LA, USA.

### Flow cytometry

Flow cytometry was used to determine the CD58 expression on HFLS-RA cells with a flurescein isothiocyanate (FITC)-labeled CD58 mouse monoclonal antibody (Catalog number: sc-81734, Santa Cruz Biotechnology, TX, USA). In 1.5-ml Eppendorf tubes, 100 μl of HFLS-RA cell suspension containing approximately 1 × 10^6^ cells suspended in PBS was added. Then, 100 μl of different FITC CD58 antibody concentrations (200, 100 and 50 μM) prepared in PBS from stock solution were added and incubated at 4°C for 1 h. After incubation, the mixture was centrifuged at 1000 rpm for 10 min, and pellets were transferred to flow cytometry tubes after resuspending with 2 ml of cold PBS. A FACSCalibur flow cytometry instrument (BD Biosciences, CA, USA) operated with CellQuest Pro software (BD Biosciences) was used, and the data were further analyzed by using the FlowJo program (v10; FlowJo, OR, USA). 10,000 cells were analyzed, and all samples were analyzed with FL1 fluorescence and side-scatter detectors for dot plots. Dot plots and histograms for cell populations according to fluorescence (FL1) were plotted for different concentrations of FITC CD58 antibody. Cells alone without FITC CD58 antibody were used as a negative control for the experiment.

### Expression of CD58 on HFLS-RA cells using microscopy

HFLS-RA cells were grown to confluency, and 10,000 cells were coated onto chamber slides. After 48 h at 37°C with 5% CO_2_, the medium was removed from each well and chilled, and enough methanol was added to cover the surface for fixing the cells. After incubating the slides for 15 min at -20°C, the methanol was removed, and PBS was used to hydrate the slides. The slides were then washed one more time with PBS. FITC-labeled CD58 mouse monoclonal antibody (Santa Cruz Biotechnology) was added to the cells and incubated for 1 h. After washing with PBS, cover slips were mounted, and the cells were visualized under a microscope. Images were taken at 40x magnification using the EVOS FL cell imaging system microscope with color CCD camera (Life Technologies, NY, USA).

### PLA

#### Step 1: Coating of cells in eight-well chamber slides

- Both T cells and HFLS-RA cells were grown to 70% confluency.
- Approximately 20,000 Jurkat cells were added to each well, and the chamber slides were incubated for 30 min at 37°C with 5% CO_2_.
- Approximately 10,000 HFLS-RA cells were treated with different concentrations of SFTI-a peptide separately for 2 h at 4°C and added over Jurkat cells that were previously coated onto the well chamber slides.
- The slides were incubated for 48 h at 37°C with 5% CO_2_.

#### Step 2: Blocking & fixing

- The medium was gently removed from the corners of the wells.
- Enough chilled methanol to cover the surface was added to each well for fixing the cells.
- After incubating the slides for 15 min at −20°C, the methanol was removed, and PBS was used to hydrate the slides.
- The slides were washed once more with PBS.
- Two drops of blocking solution was added to each well, and they were incubated for 90 min at room temperature in a moist chamber.

#### Step 3: Primary antibody addition

- The blocking solution was removed, and the slides were washed gently with PBS.
- The CD58 and CD2 (Santa Cruz Biotechnology) primary antibodies were used at a dilution of 2:100 by diluting with antibody diluent solution provided in kit (2 μl [CD58 Ab] + 2 μl [CD2 Ab] + 196 μl antibody diluent solution per well).
- After addition of primary antibody solution, the slides were incubated overnight at 4°C in a moist chamber with gentle shaking.

#### Step 4: Addition of probes

- The primary antibody solution was removed, and each well was washed very gently with 100 μl of PBS.
- The probe solution was prepared using 1:1:4 ratio of positive probe:negative probe:antibody diluent solution, respectively.
- After removing PBS, approximately 80 μl of probe solution was added to each well, which were incubated at 37°C for 1 h in a moist chamber.

#### Step 5: Addition of ligase solution

- The probe solution was discarded, and all wells were washed twice with 100 μl of wash buffer A.
- Ligase solution was prepared by mixing together 16 μl of ligation solution + 2 μl of ligase + 62 μl of high purified water.
- 80 μl of this freshly prepared ligase solution was added gently to each well, which were incubated at 37°C for 30 min in a moist chamber.

#### Step 6: Addition of amplification and polymerase solution

- All procedures from this step forward were carried out in a dark (light-protected) environment.
- After removing the ligase solution, the wells were washed gently twice with wash buffer A.
- Amplification/polymerase solution was prepared by mixing together 1 μl polymerase + 16 μl amplification (red) solution + 63 μl purified water.
- 80 μl of the above solution was added to each well in the dark, and the plates were incubated in a 37°C moist chamber in the dark for 100 min.

#### Step 7: Final washing

- Amplification/polymerase solution was removed from each well, and they were washed gently twice with 120 μl of wash buffer B.
- 0.01x wash buffer B (in phosphate-buffered saline [PBS]) was prepared, and each well was washed with this solution.
- The well on slides were removed (wells are removed after all the washing step to prepare the slide for microscopic imaging).
- Keeping the slide surface slightly wet with 0.01x wash buffer B, a small amount of mounting medium with DAPI solution was added to the slide.
- Coverslips were placed over the slides, and images were obtained by using an Olympus Fluoview 1.0 confocal microscope with DAPI and Texas Red filters.

Wash buffers A and B were provided in the kit. The composition of the buffers is not disclosed by the vendor. A bio-protocol by Lin *et al*. [22] provides the composition of Duolink *in situ* buffers A and B. The incubation time of 48 h was established by carrying out PLA at 24- and 48-h times. At 48 h, T cells and HFLS-RA cells were adherent, and washing steps did not wash away all the cells.

### Controls

Control experiments were conducted with HFLS-RA cells and T cells without addition of primary antibodies to CD2 and CD58 but with addition of secondary antibodies and PLA probes. Experiments were performed as described in steps 1–7 above.

Experiments with HFLS-RA cells and T cells were carried out as described above by adding primary and secondary antibodies only to CD58 and with PLA probes.

PLA experiments were performed with HFLS-RA cells and T cells separately without co-culture.

### Microscopy & analysis

The microscopic visualization was carried out on an Olympus Fluoview FV10i confocal microscope. DAPI (excitation 359, emission 461) and Texas Red (excitation 595, emission 612) filters were used for detection purposes at 60x zoom. For SFTI-a BODIPY conjugate, a Mg+ green (excitation 507, emission 531) filter was used. Different images were taken from each well containing different concentrations of SFTI-a and cells only. All images were taken in a single plane and at 60x magnification.

In the ImageJ software, the counting was done separately for the DAPI and the red fluorescence PLA dots for each image. Before counting, the color threshold was adjusted such that the objects were stained entirely but were distinctively highlighted. To separate overlapping objects, the settings used were: Process > Binary > Watershed. The objects were then analyzed using setting: Analyze > Analyze particles, choosing show outline option. The size range used was 0–1000 microns (by default in the software). Choosing Watershed and outline options is important to separate overlapping objects. Statistical analysis of these quantified red dots was done using SAS software (SAS Institute Inc., NC, USA). For analysis, the generalized linear model (GLM) procedure was used. The GLM procedure uses a method of least-squares to fit general linear models; it includes regression, analysis of variance, analysis of covariance, multivariate analysis of variance, and partial correlation.

### Colocalization of CD2 & CD58 using HFLS-RA & T cells

HFLS-RA cells and T cells were grown to confluency and coated onto chamber slides as described in Step 1. The cells were then fixed as described in Step 2. After the blocking solution was removed, the cells were washed with PBS and fluorescently labeled antibodies to CD2 (PE-CF594 Mouse Anti-Human CD2, BD BioSciences Catalog number: 562319) and CD58 (Santa Cruz Biotechnology, Catalog number: sc-81734) were added. Cells were incubated for 2 h at room temperature in a moist chamber with gentle shaking. The slides were washed and mounted with mounting media with DAPI. Cells without addition of any antibody were used as controls. Images were taken with Olympus BX63 fitted with deconvolution optics using DAPI, FITC and Texas Red filters at 40x magnification and processed by using CellSens dimension software.

## Results & discussion

Transient interactions between cells carried out via cell surface proteins play a key role in the immune system for generating an immune response. Protein–protein interactions between CD2 and CD58 help to stabilize and generate signaling in T-cell autoimmune response. CD2 is upregulated upon T-cell activation, and CD58 is overexpressed in antigen-presenting cells in autoimmune diseases such as RA. HFLS-RA cells derived from human RA cells provide a good model to bind to T cells. These cells are known to line the joints, producing synovial fluid. HFLS-RA cells are used to study signaling pathways that are important in the development of joint inflammation and RA [23–25]. Fibroblast synoviocytes are known to express adhesion or costimulatory molecules that help them to act as antigen-presenting cells [26]. Cell adhesion interactions between adherent and nonadherent cells have been studied using fluorescent probes [27] and PLA has been utilized to study PPI in nonadherent cells [28,29].

**Figure 2.**
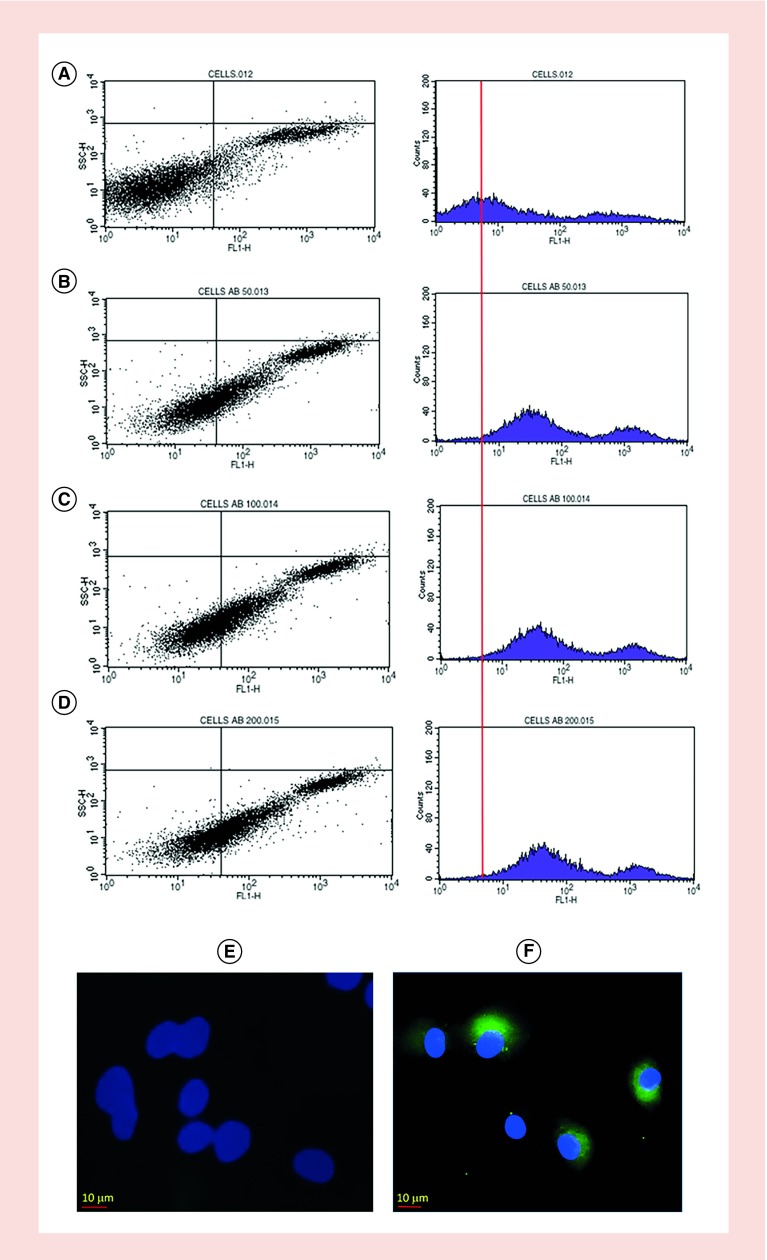
**Expression of CD58 protein on HFLS-RA cells.** **(A–D)** Verified by flow cytometry without FITC CD58 antibody and upon addition of 50, 100 and 200 μM of FITC CD58 antibody, respectively. **(E–F)** Confocal microscopy images of HFLS-RA cells labeled with FITC-CD58. Nuclei stained with DAPI (blue). HFLS-RA: Human fibroblast-like synoviocyte-rheumatoid arthritis; FITC: Flurescein isothiocyanate.

**Figure 3.**
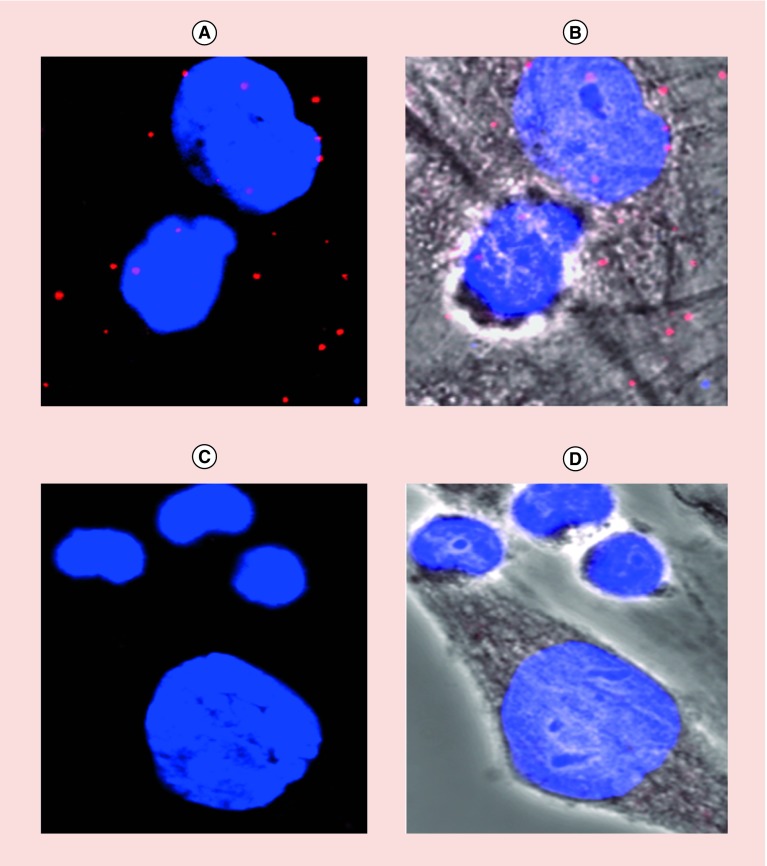
**Protein–protein interaction and its inhibition indicated by proximity ligation assay using T cells and HFLS-RA cells.** Expanded regions of **(A)** PPI without treatment showing red fluorescence dots. Nuclei are shown stained blue with DAPI. **(B)** Bright field image showing HFLS-RA cells and T cells with PLA red fluorescence dots. **(C & D)** PLA in the presence of compound SFTI-a at a concentration of 100 μM. Notice the decrease in the number of red dots, indicating PPI inhibition between CD2 and CD58. HFLS-RA: Human fibroblast-like synoviocyte-rheumatoid arthritis; PLA: Proximity ligation assay; PPI: Protein–protein interaction.

**Figure 4.**
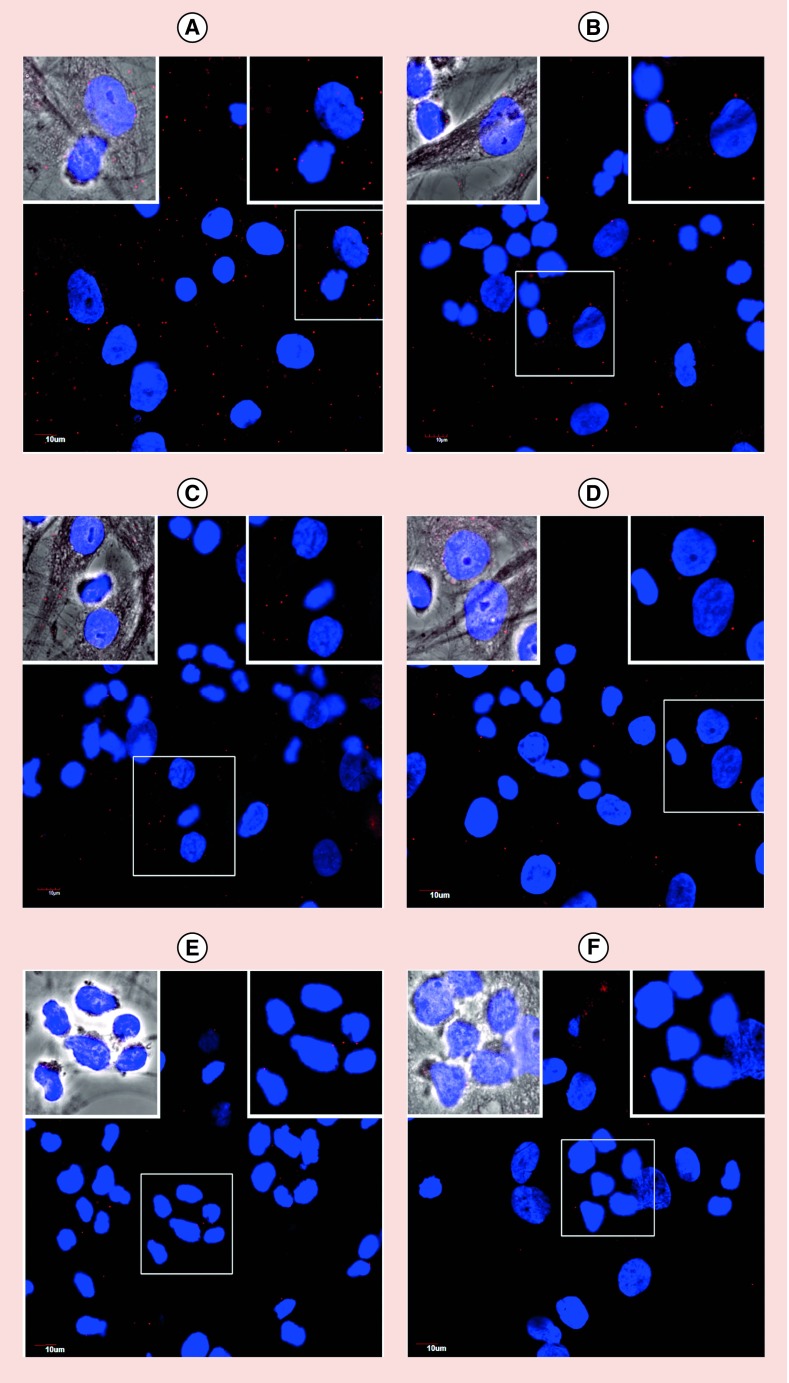

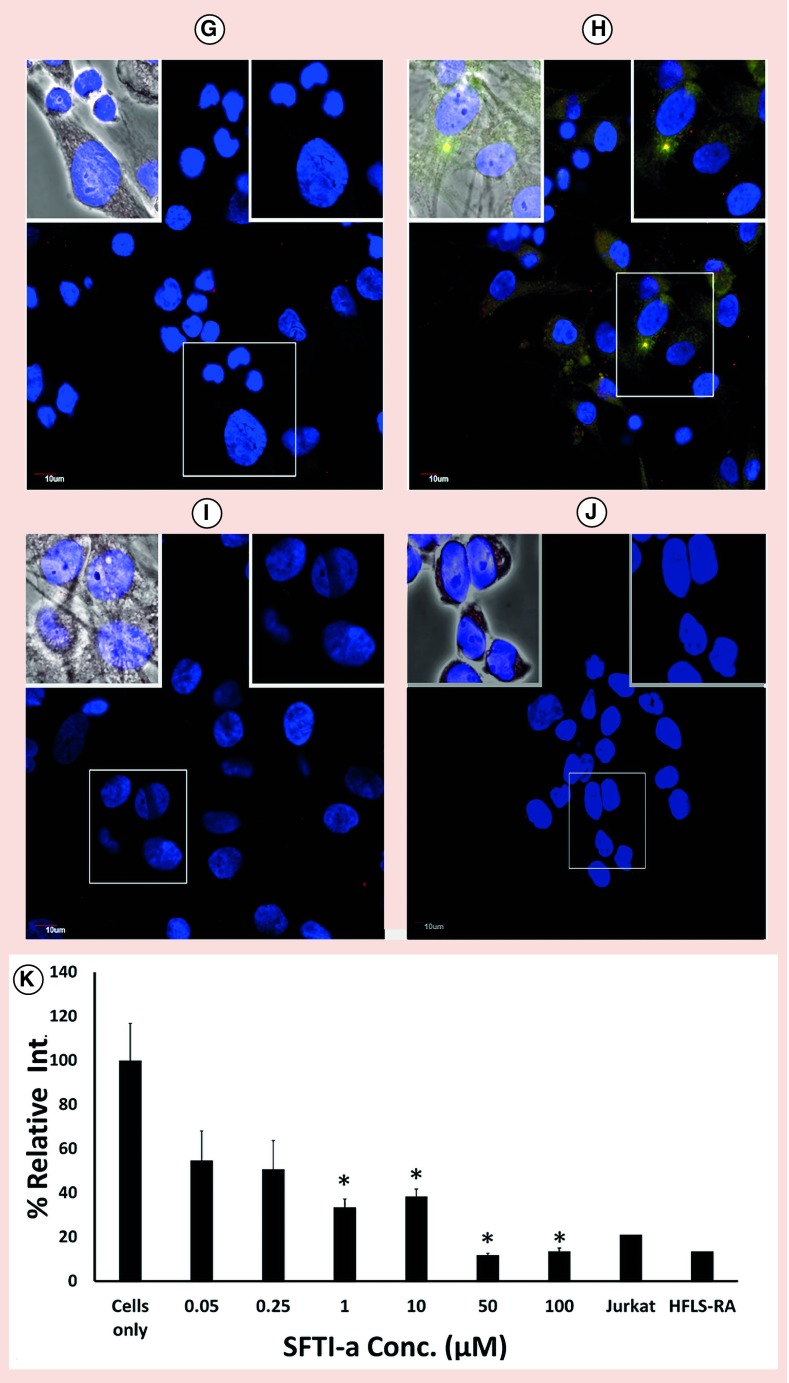
**Protein–protein interaction and its inhibition indicated by proximity ligation assay using different concentrations of SFTI-a.** **(A)** HFLS-RA and T cells in the absence of peptide showing PPI as indicated by red dots. **(B)** 50 nM, **(C)** 250 nM, **(D)** 1 μM, **(E)** 10 μM, **(F)** 50 μM, **(G)** 100 μM of SFTI-a, **(H)** BODIPY conjugate of SFTI-a. Note the green fluorescence on HFLS-RA cells due to binding of a conjugate of SFTI-a to CD58 on cells. Conjugate was also able to inhibit PPI as shown by a decrease in red fluorescence compared to control without any peptide. **(I)** HFLS-RA cells only, **(J)** T cells only, **(K)** quantification of PPI inhibition by PLA and decrease in fluorescence at different concentrations of peptide SFTI-a. Nuclei are shown stained blue with DAPI. HFLS-RA: Human fibroblast-like synoviocyte-rheumatoid arthritis; PLA: Proximity ligation assay; PPI: Protein–protein interaction.

The expression of CD58 on HFLS-RA cells was confirmed by flow cytometry and microscopy using FITC-labeled anti-CD58 that binds to the adhesion domain of CD58 (Figures 2A–F). Figure 2A shows HFLS-RA cells without fluorescently labeled CD58 antibody with most of the cells in the left lower quadrant and a histogram with cells on the left side. Upon addition of different concentrations of FITC-CD58 antibody, the cell population moved to the right side in the histogram, indicating the number of cells with FITC-CD58 labeling (Figures 2B–D). The majority of the cell population shifted to the right side of the histogram, indicating expression of CD58 on HFLS-RA cells. This was further confirmed by confocal microscopy (Figure 2E & F) where cells showed green fluorescence outside of the nucleus, indicating cell surface expression of the CD58 protein. Jurkat cells are known to express the CD2 protein. Further, we confirmed the colocalization of CD2 and CD58 using fluorescently labeled antibody to CD2 (Texas red) and CD58 (FITC) in a co-culture of HFLS-RA cells and T cells using microscopy. The presence of red and green fluorescence in the co-cultured cells on slides clearly indicates the co-localization of CD2 and CD58 (Supplementary data). To study the interactions of CD2 and CD58 from T cells and HFLS-RA cells, the PLA was employed. One of the hurdles faced in the development of the assay was a problem with nonadherent Jurkat cells. The PLA involves several steps of washing, and a nonadherent cell will create a problem because several washing steps in which a number of nonadherent cells wash away is significant. To overcome this problem, we incubated the Jurkat cells first on chamber well plates and added HFLS-RA cells, which are adherent cells. Incubating HFLS-RA cells with Jurkat cells helped the Jurkat cells to immobilize during washing. Thus, we were successfully able to evaluate the PPI by PLA with the use of two different cell lines. When T cells were incubated with HFLS-RA cells without any peptide, SFTI-a treatment and PLA was performed, there were number of red dots (Figures 3 & 4) of fluorescence due to CD2–CD58 interactions. After treatment with 100 μM SFTI-a peptide, there was a significant decrease in red dots (Figures 3C & D), indicating PPI inhibition. Cells that were incubated with different concentrations of SFTI-a peptide showed a significant decrease in bright red spots. SFTI-a showed dose-dependent inhibition of the interaction between CD2 and CD58 proteins present on Jurkat cells and HFLS-RA cells, respectively (Figure 4B–G). As controls, the PLA was carried out on individual cells (T cells and HFLS-RA cells) as well as co-culturing the HFLS-RA cells and T cells and adding primary and secondary antibodies to only HFLS-RA cells and T cells (Figure 4I & J). Control experiments when visualized under a microscope did not show any red fluorescence dots, indicating the specificity of the assay. These results indicate that PPI can be detected between the two cells as shown by red fluorescence using PLA assay. Inhibition of PPI between the two cells can be visualized using microscopy. Furthermore, inhibition of PPI can be quantified. The number of fluorescence red dots was quantified using ImageJ software, and the data (Figure 4K) show a clear inhibition of PPI by SFTI-a peptide. The data generated from the experiment were analyzed by SAS software with GLM code. The statistical analysis indicated that the PPI inhibition observed with all concentrations of SFTI-a peptide (10, 50 and 100 μM) was significant in comparison with that of untreated wells with p < 0.0001 (Figure 4K). SFTI-a conjugate is a fluorescently labeled peptide with BODIPY attachment. The experiment with SFTI-a conjugate treatment showed that SFTI-a peptide was bound to the cell surface of HFLS-RA cells as shown by green fluorescence of BODIPY (Figure 4H). Thus, the peptide and conjugate bind to CD58 on the cell surface and inhibit the interaction between Jurkat cells and HFLS-RA cells. This was determined by significant inhibition of the interactions of CD2 and CD58 proteins (decrease in bright red spots) present on different cells (Figure 4K). The experiment indicated that in even lower concentrations of SFTI-a (50 and 250 nM), there was a significant reduction in a number of red dots compared with control (p < 0.001).

Overall, we employed the PLA to study PPI between two cell surface proteins on different cells. We have carried out this assay on floating and adherent cells. It is particularly useful to study immune cells, as the interaction between immune cells involves several different proteins. The assay can also be used to study PPI between adherent cells as well as PPI between cells if the cells’ transiently express proteins on the cell surface.

### Pitfalls & practical solutions

Jurkat cells are floating cells, and there can be seven or more washing steps in PLA. Hence, the number of Jurkat cells needed is relatively large.

Washing steps must be done carefully, as adhesion interaction between immune cells is relatively weak, and it is possible to wash away most of the cells used for the study.

## References

[1] Stumpf MP, Thorne T, de Silva E (2008). Estimating the size of the human interactome. *Proc. Natl Acad. Sci. USA*.

[2] Sperandio O, Reynes CH, Camproux AC, Villoutreix BO (2010). Rationalizing the chemical space of protein–protein interaction inhibitors. *Drug Discov. Today*.

[3] Wells JA, McClendon CL (2007). Reaching for high-hanging fruit in drug discovery at protein–protein interfaces. *Nature*.

[4] Arkin MR, Tang Y, Wells JA (2014). Small-molecule inhibitors of protein–protein interactions: progressing toward the reality. *Chem. Biol.*.

[5] Long EO (2011). ICAM-1: getting a grip on leukocyte adhesion. *J. Immunol.*.

[6] Kanthala S, Banappagari S, Gokhale A (2015). Novel peptidomimetics for inhibition of HER2:HER3 heterodimerization in HER2-positive breast cancer. *Chem. Biol. Drug. Des.*.

[7] Kanthala SP, Liu YY, Singh S, Sable R, Pallerla S, Jois SD (2017). A peptidomimetic with a chiral switch is an inhibitor of epidermal growth factor receptor heterodimerization. *Oncotarget*.

[8] Sable R, Durek T, Taneja V (2016). Constrained cyclic peptides as immunomodulatory inhibitors of the CD2:CD58 protein–protein interaction. *ACS Chem. Biol.*.

[9] Wang JH, Smolyar A, Tan K (1999). Structure of a heterophilic adhesion complex between the human CD2 and CD58 (LFA-3) counterreceptors. *Cell.*.

[10] Hall RA, George SR, O'Dowd BF, Sibley DR (2005). Co-immunoprecipitation as a strategy to evaluate receptor–receptor or receptor–protein interactions. *G Protein-Coupled Receptor–Protein Interactions*.

[11] Soderberg O, Gullberg M, Jarvius M (2006). Direct observation of individual endogenous protein complexes *in situ* by proximity ligation. *Nat. Methods*.

[12] Roussis IM, Guille M, Myers FA, Scarlett GP (2016). RNA whole-mount *in situ* hybridisation proximity ligation assay (rISH–PLA), an assay for detecting RNA–protein complexes in intact cells. *PLoS One*.

[13] Trifilieff P, Rives ML, Urizar E (2011). Detection of antigen interactions *ex vivo* by proximity ligation assay: endogenous dopamine D2–adenosine A2A receptor complexes in the striatum. *Biotechniques*.

[14] Fichter CD, Timme S, Braun JA (2014). EGFR, HER2 and HER3 dimerization patterns guide targeted inhibition in two histotypes of esophageal cancer. *Int. J. Cancer*.

[15] Fredriksson S, Gullberg M, Jarvius J (2002). Protein detection using proximity-dependent DNA ligation assays. *Nat. Biotechnol.*.

[16] Lee KH, Holdorf AD, Dustin ML, Chan AC, Allen PM, Shaw AS (2002). T cell receptor signaling precedes immunological synapse formation. *Science*.

[17] Zal T, Zal MA, Gascoigne NR (2002). Inhibition of T cell receptor–coreceptor interactions by antagonist ligands visualized by live FRET imaging of the T-hybridoma immunological synapse. *Immunity*.

[18] Chen L, Flies DB (2013). Molecular mechanisms of T cell co-stimulation and co-inhibition. *Nat. Rev. Immunol.*.

[19] Davis SJ, Ikemizu S, Evans EJ, Fugger L, Bakker TR, van der Merwe PA (2003). The nature of molecular recognition by T cells. *Nat. Immunol.*.

[20] Raychaudhuri S, Thomson BP, Remmers EF (2009). Genetic variants at CD28, PRDM1 and CD2/CD58 are associated with rheumatoid arthritis risk. *Nat. Genet.*.

[21] Uppal T, Bhupathiraju NDK, Vicente MGH (2013). Synthesis and cellular properties of Near-IR BODIPY–PEG and carbohydrate conjugates. *Tetrahedron*.

[22] Lin MZ, Martin JL, Baxter RC (2015). Proximity ligation assay (PLA) to detect protein–protein interactions in breast cancer cells. *Bio-protocol*.

[23] Jones DS, Jenney AP, Swantek JL, Burke JM, Lauffenburger DA, Sorger PK (2017). Profiling drugs for rheumatoid arthritis that inhibit synovial fibroblast activation. *Nat. Chem. Biol.*.

[24] Chen H, Pan J, Wang JD, Liao QM, Xia XR (2016). Suberoylanilide hydroxamic acid, an inhibitor of histone deacetylase, induces apoptosis in rheumatoid arthritis fibroblast-like synoviocytes. *Inflammation*.

[25] Zhang Y, Zhang B (2016). Trichostatin A. An inhibitor of histone deacetylase, inhibits the viability and invasiveness of hypoxic rheumatoid arthritis fibroblast-like synoviocytes via PI3K/Akt signaling. *J. Biochem. Mol. Toxicol.*.

[26] Lambert N, Lescoulie PL, Yassine-Diab B (1998). Substance P enhances cytokine-induced vascular cell adhesion molecule-1 (VCAM-1) expression on cultured rheumatoid fibroblast-like synoviocytes. *Clin. Exp. Immunol.*.

[27] Liu J, Chow VT, Jois SD (2004). A novel, rapid and sensitive heterotypic cell adhesion assay for CD2–CD58 interaction, and its application for testing inhibitory peptides. *J. Immunol. Methods*.

[28] Debaize L, Jakobczyk H, Rio A-G, Gandemer V, Troadec M-B (2017). Optimization of proximity ligation assay (PLA) for detection of protein interactions and fusion proteins in non-adherent cells: application to pre-B lymphocytes. *Mol. Cytogenet.*.

[29] David L, Gokhale A, Jois S (2016). CD74/DQA1 dimers predispose to the development of arthritis in humanized mice. *Immunology*.

