# Supplementary material for: Proximity ligation assay to study protein–protein interactions of proteins on two different cells
Source: Biotechniques. 2018 Sep 19;65(3):149–57. doi: 10.2144/btn-2018-0049 (PMC6770473; doi:10.2144/btn-2018-0049)
Supplement: Supplementary file 1 [file btn-65-149-s1.docx]

**Supporting Information**

**
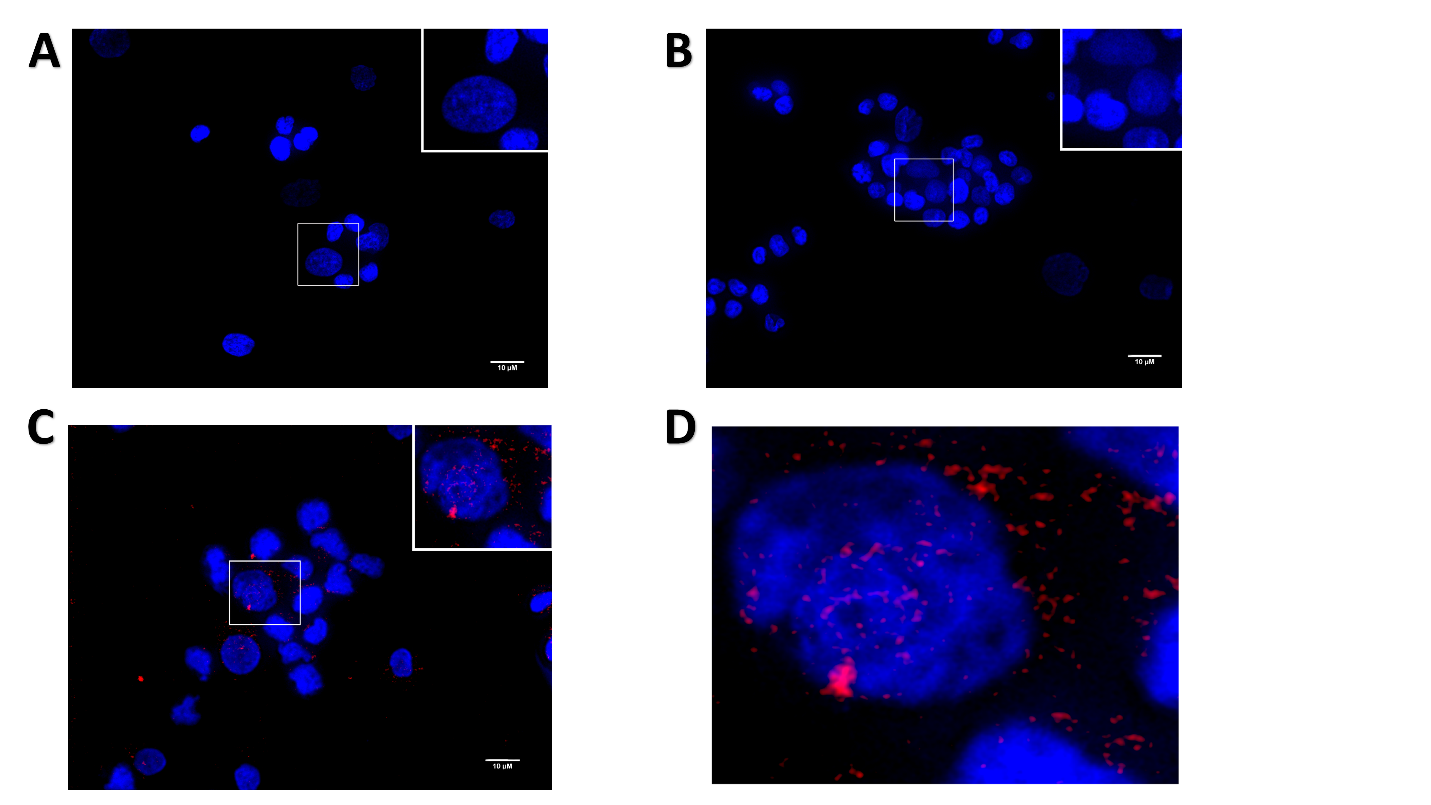
**

**Figure 1. Control experiments for protein-protein interactions of CD2-CD58 and its detection using PLA assay**. HFLS-RA and T-cells (**A**) in the absence of both primary antibodies; (**B**) in the presence of only CD2 antibody; and (**C**) in the presence of both antibodies illustrating PLA red fluorescence dots. **D**) expanded region of positive PLA images showing red fluorescence due to protein-protein interactions. Nuclei stained with DAPI (blue). Slides were visualized under the microscope (Olympus BX63 fitted with deconvolution optics), and images were taken at 40× by using CellSens Dimension software. Scale bar = 10 μm

**
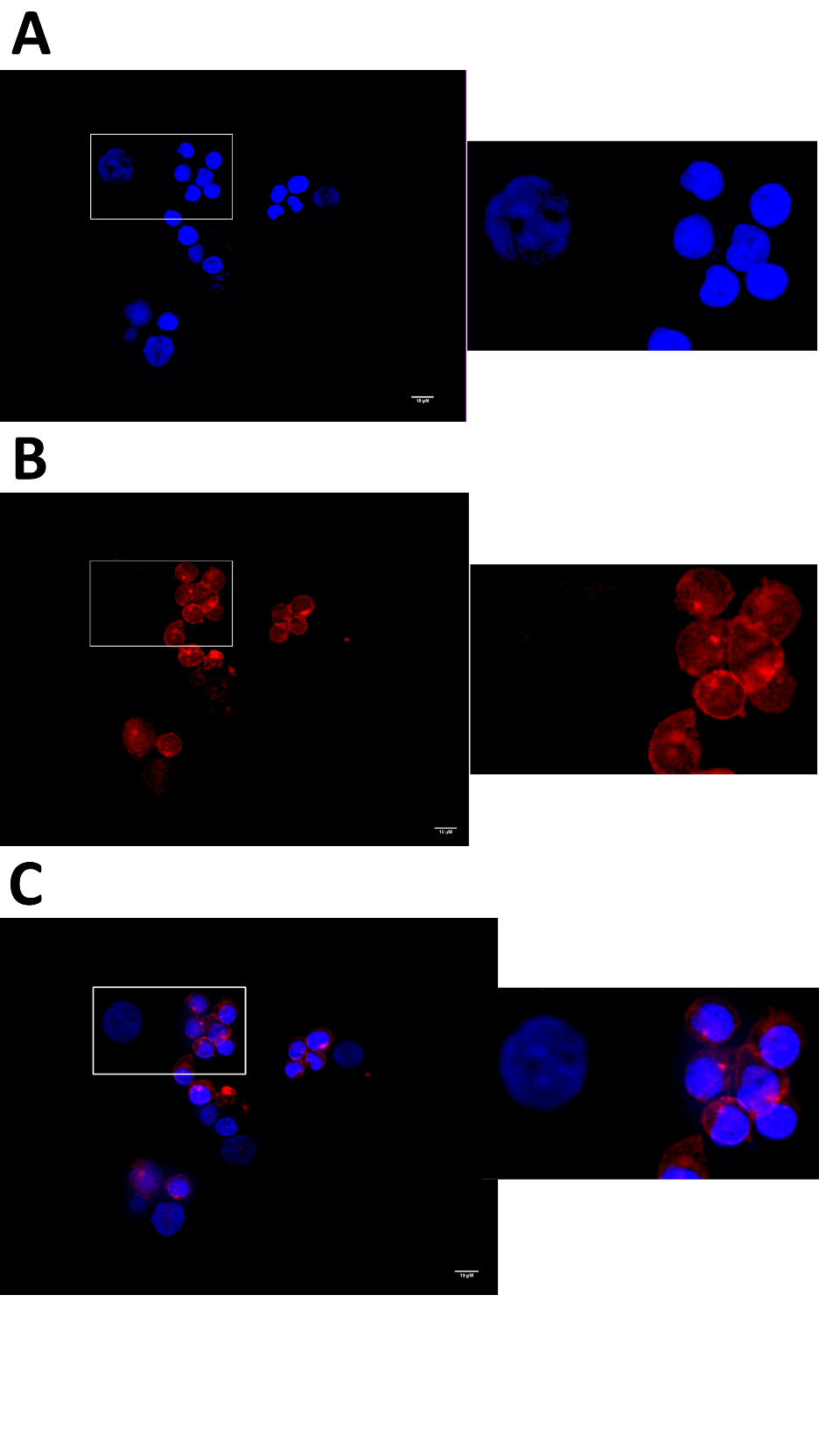
**

**
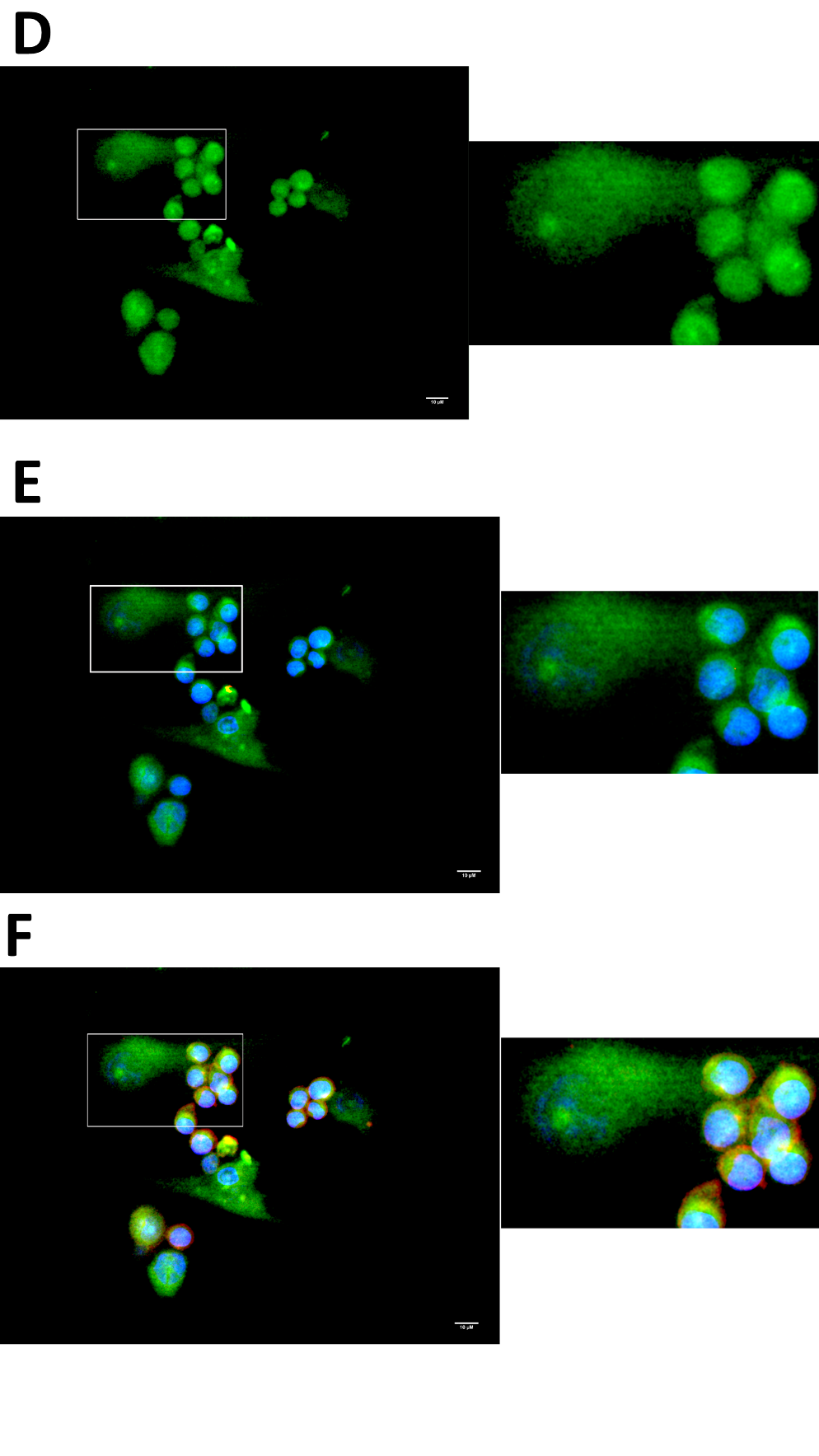
**

**Figure 2**. **Colocalization of CD2 and CD58 on HFLS-RA cells and T cells during adhesion.** Expanded regions are shown on the right side of each image for clarity. **A**) HFLS-RA cells and Jurkat cells. Nuclei are shown stained blue with DAPI staining. **B**) HFLS-RA cells and Jurkat cells. CD2 stained with Texas red-Ab. **C**) HFLS-RA cells and Jurkat cells. Overlapped images of A and B showing cells with nucleus and CD2 staining. **D**) HFLS-RA and T cells. CD58 stained with FITC-Ab. **E**) overlapped images of A and D showing nucleus and CD58 staining. **F**) HFLS-RA and T cells showing colocalization of CD2 (red), CD58 (green) and nucleus (blue). Merged green and red stains are shown as slight yellow indicating colocalization of CD2 and CD58. Slides were visualized under the microscope (Olympus BX63 fitted with deconvolution optics), and images were taken at 40× by using CellSens Dimension software. Scale bar = 10 μm
